# The Relationship between Alcohol Drinking Patterns and Sleep Duration among Black and White Men and Women in the United States

**DOI:** 10.3390/ijerph15030557

**Published:** 2018-03-20

**Authors:** Chandra L. Jackson, Symielle A. Gaston, Rui Liu, Kenneth Mukamal, Eric B. Rimm

**Affiliations:** 1 Epidemiology Branch, National Institute of Environmental Health Sciences, National Institutes of Health, Department of Health and Human Services, 111 TW Alexander Drive, Research Triangle Park, NC 27709, USA; symielle.gaston@nih.gov; 2 Social & Scientific Systems, Inc., Research Triangle Park, NC 27703, USA; RLiu2@s-3.com; 3 Department of Medicine, Beth Israel Deaconess Medical Center, Boston, MA 02215, USA; kmukamal@bidmc.harvard.edu; 4 Nutrition Department, Harvard T.H. Chan School of Public Health, Boston, MA 02215, USA; erimm@hsph.harvard.edu; 5 Department of Epidemiology, Harvard T.H. Chan School of Public Health, Boston, MA 02215, USA; 6 Channing Division of Network Medicine, Brigham and Women’s Hospital and Harvard Medical School, Boston, MA 02215, USA

**Keywords:** alcohol drinking, sleep, sleep deprivation, sleep initiation and maintenance disorders, health status disparities, minority health, sex

## Abstract

In the United States, racial minorities generally experience poorer cardiovascular health compared to whites, and differences in alcohol consumption and sleep could contribute to these disparities. With a nationally representative sample of 187,950 adults in the National Health Interview Survey from 2004 to 2015, we examined the relationship between alcohol-drinking patterns and sleep duration/quality by race and sex. Using Poisson regression models with robust variance, we estimated sex-specific prevalence ratios for each sleep duration/quality category among blacks compared to whites within categories of alcohol-drinking pattern, adjusting for socioeconomic status and other potential confounders. Across alcohol drinking patterns, blacks were less likely than whites to report recommended sleep of 7–<9 h/day. Short (PR = 1.30 [95% CI: 1.22–1.39]) and long (PR = 1.30 [95% CI: 1.07–1.58]) sleep were 30% more prevalent among black-male infrequent heavy drinkers compared to white-male infrequent heavy drinkers. Short (PR = 1.27 [95% CI: 1.21–1.34]) sleep was more prevalent among black-female infrequent heavy drinkers compared to white-female infrequent heavy drinkers, but there was no difference for long sleep (PR = 1.09 [95% CI: 0.97–1.23]). Black female infrequent moderate drinkers, however, had a 16% higher (PR = 1.16 [95% CI: 1.01–1.33]) prevalence of long sleep compared to their white counterparts. Environmental, social, and biological factors contributing to these findings, along with their impact on disparate health outcomes, should be studied in greater detail.

## 1. Introduction

According to the National Survey on Drug Use and Health, more than half of US adults aged 26 years and older were current alcohol users in 2016 [1]. Moderate alcohol consumption (1–2 drinks/day for men and 1 drink/day for women) may have a protective effect against selected health-related outcomes, including cardiovascular disease (CVD) and all-cause mortality, albeit with potential differences by age and race [2,3,4,5]. However, a recent systematic review suggests that moderate-to-excessive alcohol consumption (≥14 drinks/week for men and ≥7 drinks/week for women) is clearly associated with increased risk for deleterious acute health outcomes like injury and mortality [6]. Alcohol consumption can also negatively impact sleep, and while 7–8 h of sleep is typically associated with better cardiovascular health outcomes, insufficient sleep is positively associated with CVD risk factors like weight gain, diabetes, and hypertension [7,8].

The relationship between alcohol consumption and sleep is complex. Common alcohol-related sleep disorders include symptoms like poor sleep quality, sleep disturbances, and short sleep duration [9,10,11,12,13,14,15,16,17,18,19]. Individuals who suffer from sleep disorders such as insomnia tend to self-medicate and use alcohol to initiate sleep, making alcohol the most common over-the-counter sleep aid [16]. The general public may lack awareness of the potential harms associated with using alcohol to initiate sleep [20,21]. Previous studies, however, suggest that perpetual use of alcohol as a sleep aid is counterproductive, disrupts sleep, and intensifies the need to consume more alcohol [19,22]. Furthermore, alcohol consumption can degrade sleep quality when used in large amounts for extended periods [22]. For instance, high alcohol consumption is associated with decreases in rapid-eye movement (REM) sleep, sleep continuity, sleep latency (time to fall asleep), and sleep duration [11]. The effects of alcohol on sleep architecture may remain even after cessation of alcohol consumption. Prior literature has shown that compared to non-alcoholic adults, alcoholic adults’ post-alcohol withdrawal exhibited differences in sleep architecture like decreased deep sleep (or slow wave sleep) [14]. 

Associations between alcohol use and sleep have been shown to also vary by race/ethnicity. In an analysis of objective sleep patterns among alcoholic (met DSM-IV criteria for alcohol dependence without major pre-existing psychiatric disorders) and non-alcoholic individuals using polysomnographic and spectral sleep electroencephalography (EEG) measures, alcoholic individuals had more sleep abnormalities (i.e., reduced sleep efficiency, longer sleep latency) than controls [23]. Furthermore, black male alcoholic individuals had more severe sleep abnormalities including longer sleep latency and lower deep sleep than their white counterparts [23]. Although racial differences were observed between Black and White men, to our knowledge, there is no comparable research, to date, among women. 

Further investigation of race-related differences is warranted among both sexes. Racial variations in genetic polymorphisms associated with ethanol metabolism could contribute to racial differences in alcohol consumption as well as acute responses to alcohol [24,25,26]. Racial and sex differences in the social and physical environments also influence drinking patterns, health behaviors including sleep, and alcohol-associated problems [27,28,29]. For example, black Americans experience greater chronic stress, on average, due to a greater likelihood of encountering environmental stressors daily (e.g., poverty, discrimination, suboptimal residential environments) [29], which could exacerbate differences in sleep quality and alcohol consumption [23]. To our knowledge, there is no research comparing the impact of alcohol drinking patterns on sleep between blacks and whites. Our study objectives were to determine whether there are black-white and sex differences in the relationship between alcohol drinking patterns and measures of sleep duration and sleep quality. 

## 2. Materials and Methods

### 2.1. The National Health Interview Survey

We analyzed data from the National Health Interview Survey (NHIS), a series of cross-sectional, nationally representative surveys that use a three-stage stratified cluster probability sampling design to conduct in-person interviews in the households of non-institutionalized US civilians. A detailed description of NHIS procedures has been previously published [30]. Briefly, a probability sample of households was interviewed by trained interviewers from the US Census Bureau to obtain information about health and sociodemographic characteristics of each member of the sampled household on a continuous basis each week. The data were collected using computer-assisted personal interviewing (CAPI). A randomly selected adult and child (if present; not included in the current analysis) provided more specific health-related information. The response rate for sample adults was 80.0% (survey year range: 74.2–83.7%). The National Institute of Environmental Health Sciences’ Institutional Review Board waived approval for publicly-available, secondary data with no identifiable information, and the NHIS received informed consent from each study participant. 

### 2.2. Study Participants

Participants in these analyses included self-identified Non-Hispanic white or Non-Hispanic black (hereafter, white and black) adults aged ≥18 years. Participants were excluded if they were born outside the US or had missing data on either alcohol consumption or sleep duration. We excluded non-US born participants because evidence suggests that sleep patterns among US immigrants differ from those among individuals born in the United States [31]. Our final analytical sample comprised 187,950 adults (Supplemental Figure S1). 

### 2.3. Measures

#### 2.3.1. Alcohol Drinking Patterns 

All adults were asked about their lifetime alcohol consumption by responding to the following question: “In your entire life, have you had at least 12 drinks of any type of alcoholic beverage?” The standard size drink typically includes a 12 fl oz. bottle/can of beer, 8–9 fl oz. malt liquor, 5 fl oz. glass of wine, and 1.5 fl oz. shot of 80-proof spirits [32]. Only participants who acknowledged drinking in the past year were further queried: “In the past year, on those days that you drank alcoholic beverages, on the average, how many drinks did you have?” Interviewers defined ‘alcoholic beverages’ as including “liquor such as whiskey or gin, beer, wine, wine coolers, and any other type of alcoholic beverage”. For participants reporting consumption of at least 12 drinks in their lifetime, we combined a variable for ‘average number of drinks on days drank (coded 1, 2, 3 or more)’ with a variable for ‘days per week drank in the past year (coded ‘did not drink’ vs. ‘1–2 days’ vs. ‘3 or more days’).

Participants were placed into five mutually exclusive alcohol categories, based on their lifetime consumption patterns, as well as consumption patterns in the past year. ‘Never drinkers’ were defined as those who reported consuming <12 drinks during their lifetime. U.S. government dietary guidelines for 2015–2020 recommend no more than 1 drink per day for women and 2 for men [33]. Based on these guidelines and prior literature [34], the remaining participants were placed into the following sex-specific categories: for men, (1) 1–2 drinks/day on ≤2 days/week, (2) 1–2 drinks/day on 3–7 days/week, (3) 3+ drinks/day on ≤2 days/week, (4) 3+ drinks on 3–7 days/week; for women: (1) 1 drink/day on ≤2 days/week, (2) 1 drink/day on 3–7 days/week, (3) 2+ drinks/day on ≤2 days/week, (4) 2+ drinks/day on 3–7 days/week. Thus, for these 4 groupings, regardless of sex, participants in the first 2 categories were following recommended guidelines, participants in the second 2 exceed guidelines, and within this split were differences in number of days of consumption per week.

#### 2.3.2. Sleep Duration and Quality

Sampled adults reported how many hours they sleep, on average, in a 24-h period. Participants were instructed to report the hours of sleep in whole numbers, rounding up values of ≥30 min to the next nearest hour and rounding down values <30 min to the nearest hour. We categorized sleep duration into three groups: short (<7 h), recommended (7–<9 h), and long (≥9 h). Habitual sleep duration of 7–<9 h was used as the reference because it has been associated with the lowest levels of morbidity and mortality [7]. These measures of sleep duration were available for 2004–2015. Additionally, several measures of sleep quality were assessed by asking about “trouble falling asleep” (1–7 or more times vs. never), “trouble staying asleep” (1–7 or more times vs. never), and “waking up most days feeling rested” (‘most’ [4–7 days] vs. ‘few/none’ [0–3 days]). Sleep medication use was also assessed by asking about “sleep medication one or more times to help fall asleep or stay asleep” (1–7 or more times vs. never). All measures were based on the previous week of the survey. 

#### 2.3.3. Race/Ethnicity

Participants were asked, “What race or races do you consider yourself to be?” They then self-identified as one or more of the following categories: American Indian/Alaskan native, Asian, black/African American, white, or multiple races. Our analysis focuses on blacks/African Americans and non-Hispanic whites because the underlying biological (not necessarily genetic) and social mechanisms leading to differences in sleep duration are likely to vary by race/ethnicity [35].

#### 2.3.4. Covariates 

Based on prior literature, we included measures potentially associated with alcohol consumption, sleep duration and quality, sex, and race/ethnicity including socioeconomic status indicators, health behaviors, medical conditions, and clinical characteristics [27,28,29,34,35,36]. Educational attainment was categorized as less than high school (no high school diploma), high school (high school or general equivalency diploma), some college, and at least a college-level education (Bachelor’s degree) or greater. We categorized participants as employed or not based on employment status in the week prior to the interview, which was originally categorized as “working for pay”, “working without pay”, “job not at work”, “unemployed”, or “not in the labor force”. A dichotomized variable of unemployed/not in workforce was used versus working for/without pay. Annual household income was dichotomized as below $35,000 versus $35,000 or above, and poverty status was based on being below the 100% federal poverty level after the participants’ best estimates of total income of all family members from all sources before taxes. Marital status was categorized as married/living with partner, divorced/separated/widowed, or never married. Smoking status was categorized as ‘never’, ‘former’ or ‘current’. Leisure-time physical activity was classified as ‘none/unable’, ‘low’, or ‘high’. Participants who engaged in at least some level of activity and who provided a specific number of activity bouts were dichotomized at the midpoint of these bouts and classified as ‘low’ or ‘high’. Participants reporting ‘never’ or ‘unable to do this type activity’ were categorized as ‘never/unable’. We classified participants as having hypertension if they reported ever being told by a doctor or other health professional that they had hypertension. If a doctor or other health professional ever diagnosed them as having coronary heart disease or any kind of heart condition or disease, we combined these variables to adjust for heart disease. Sadness in the last 30 days was dichotomized as all or most of the time versus none/little/some. Self-reported height and weight were used to calculate BMI by dividing measured weight in kilograms by height in meters squared. Obesity was defined as BMI ≥ 30 kg/m^2^, overweight as 25.0–29.9 kg/m^2^, normal weight as 18.5–24.9 kg/m^2^, and underweight as BMI < 18.5 kg/m^2^ [37]. Self-reported general health status was categorized as excellent/very good, good, or fair/poor. 

### 2.4. Statistical Analysis

We pooled 12 survey years (2004–2015) of NHIS data merged by the Integrated Health Interview Series [38]. For all analyses, we used sampling weights to account for the unequal probabilities of selection resulting from the sample design, nonresponse, and oversampling of certain subgroups. Standard errors or variance estimates were calculated by using Taylor series linearization. Stata, version 14, software (StataCorp LP, College Station, TX, USA) was used for all analyses. A two-sided *p*-value < 0.05 was considered statistically significant. 

Separately for men and women, we compared the prevalence of certain sleep duration and quality categories among blacks compared to whites across categories of alcohol consumption for pre-specified sociodemographic, self-reported medical history, as well as health and behavioral characteristics using Rao-Scott second-order corrected Pearson statistics [39]. Categorical variables were reported using unweighted frequencies accompanied by the weighted percentages, with all percentages standardized to the age structure of the 2010 Census. 

Using Poisson regression with a robust variance estimator, we calculated adjusted prevalence ratios comparing blacks and whites on sleep duration and sleep quality across sex-specific categories of alcohol consumption. All models were adjusted for pre-specified demographic, socioeconomic, health behavior, and clinical characteristics, including age, BMI, educational attainment, employment status, annual household income, smoking status, physical activity, diabetes, hypertension, heart disease, cancer, feeling sad most or all the time in the past 30 days, health status, and region of residence. White participants were used as the reference for the black-white comparisons for greater statistical stability since this group had the largest sample size.

By including an interaction term for race and alcohol in sex-specific regression models, we tested for a potential interaction between alcohol consumption and sleep by race. To further test for potential interaction by sex, we included an interaction term for sex and alcohol consumption in the race-specific regression models. Since alcohol consumption recommendations differ by sex, we first collapsed the sex-specific alcohol variable into 3 categories (never, moderate, heavy) and then generated an overall 3-category variable for alcohol consumption. We included this new 3-category alcohol variable in the interaction term and then in the regression model. Finally, we assessed a potential nonlinear relation between weekly alcohol consumption (continuous variable) and sleep duration (continuous), stratified by short, recommended, and long sleep duration, using restricted cubic spline regression. The splines were generated using PROC SURVEYREG to fit the regression model, adjusting for age, BMI, educational attainment, employment status, smoking status, physical activity, region of residence, and annual household income, while using sampling weights to account for the structure of the NHIS survey data.

## 3. Results

### 3.1. Study Population Characteristics 

Our sample consisted of 187,950 (18% black and 55% women) participants (Supplemental Figure S1). Among men, 14% of white men and 27% of black men were never drinkers (Table 1). Among women, 23% of white women and 43% of black women reported never consuming alcohol (Table 2). Compared with blacks, whites were older, less likely to live in poverty and more likely to be married, to have a college education or above, to report habitually getting the recommended amount of sleep, and to engage in a high level of leisure-time physical activity across all levels of alcohol consumption. 

### 3.2. Black-White Differences in Sleep Behaviors Across Categories of Alcohol Consumption

#### 3.2.1. Black-White Differences Prior to Adjustment 

**Figure 1:**
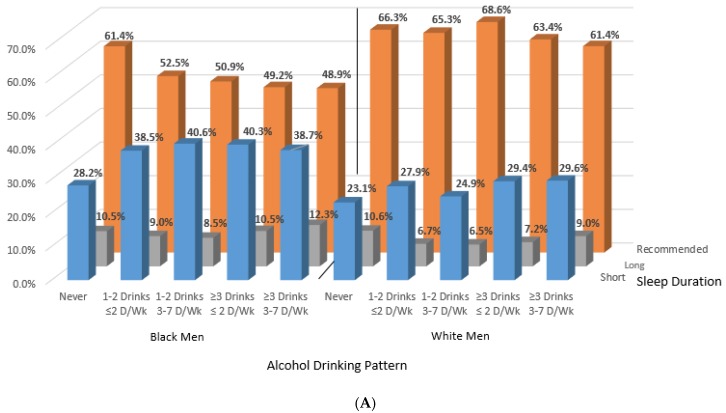

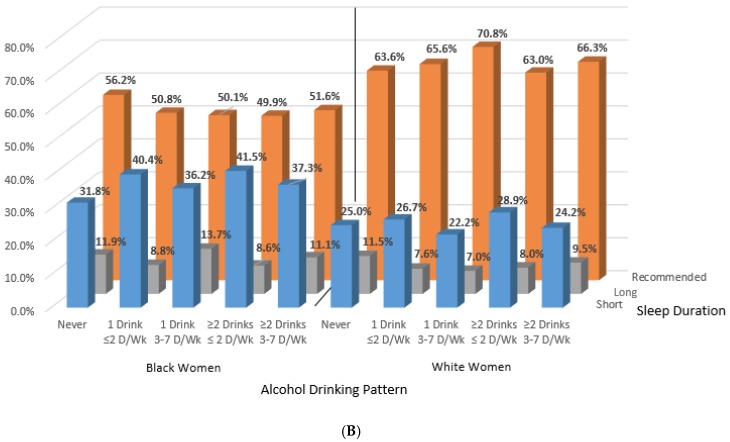
Age-standardized proportions across alcohol drinking pattern for men (**A**) and women (**B**) by race and sleep duration categories. Note: Alcohol drinking pattern for men: never = never drinkers; moderate infrequent = 1–2 drinks ≤ 2 days/week; moderate frequent = 1–2 drinks 3–7 days/week; heavy infrequent =≥ 3 drinks ≤ 2 days/week; heavy frequent =≥ 3 drinks 3–7 days/week. Sleep duration: short =< 7 h; long =≥ 9 h, recommended = 7–<9 h. Alcohol drinking pattern for women: never = never drinkers; moderate infrequent = 1 drink ≤ 2 days/week; moderate frequent = 1 drink 3–7 days/week; heavy infrequent =≥ 2 drinks ≤ 2 days/week; heavy frequent =≥ 2 drink 3–7 days/week. Sleep duration: short: <7 h; long: ≥9 h, recommended: 7–<9 h.

**Figure 2:**
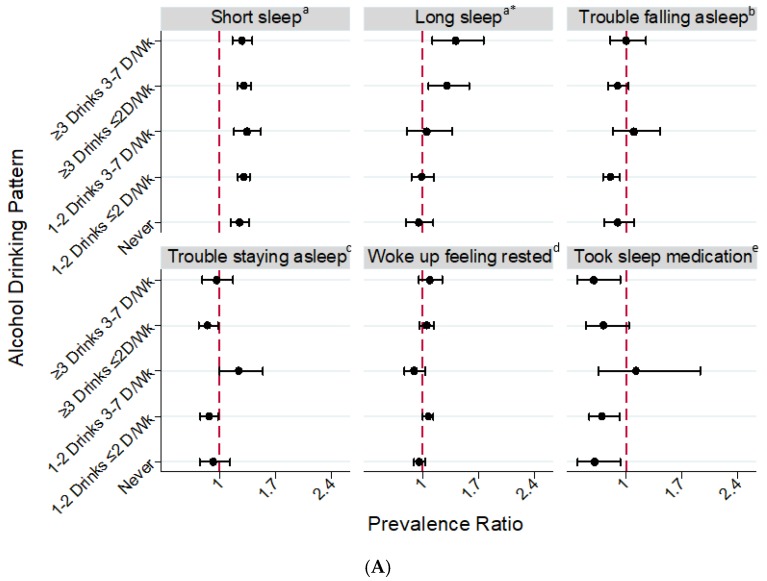

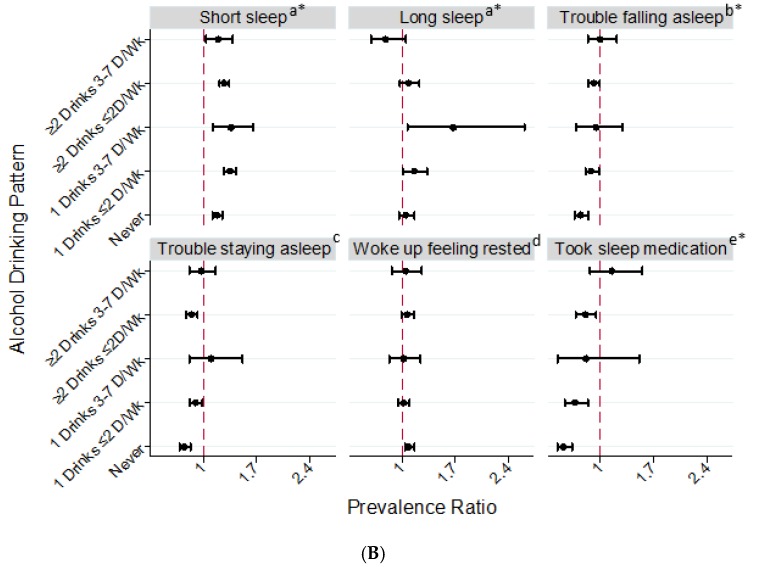
Fully-Adjusted Prevalence Ratios for Sleep Duration and Quality in Relation to Alcohol Drinking Patterns among U.S. Black Men (**A**) and Women (**B**) (referent: White Men or White Women), National Health Interview Survey, 2004–2015. Note. Short sleep: <7 h; long sleep: ≥9 h. Prevalence ratios adjusted for age, BMI, educational attainment, income, employment status, smoking, physical activity, diabetes, hypertension, heart disease, cancer, feeling sad (past 30 days), health status, and region of residence. Sleep quality data is from 2013–2015. ^a^ The referent category is white men (A) or white women (B) with recommended sleep (7–<9 h); ^b^ Number of times having trouble falling asleep over the past week (1–7 or more times vs. never); ^c^ Number of times having trouble staying asleep over the past week (1–7 or more times vs. never); ^d^ Days woke up feeling rested over the past week (4–7 days vs. 0–3 days); ^e^ Number of times taking medication for sleep over the past week (1–7 or more days vs. never). * (A) Significant interactions between race and alcohol drinking pattern for men (long sleep: *p*_interaction_ = 0.03); (B) Significant interactions between race and alcohol drinking pattern for women (short sleep: *p*_interaction_ = 0.0004; long sleep: *p*_interaction_ = 0.01; trouble falling asleep: *p*_interaction_ = 0.05; took sleep medication: *p*_interaction_ = 0.0003).

We observed black-white differences in sleep duration and quality for both men and women across alcohol consumption patterns (Figure 1 and Figure 2). The proportion of black men and women with short sleep duration increased with any alcohol consumption, but short sleep duration varied across alcohol consumption categories among Whites (Figure 1). For white men and women, those who consumed alcohol in moderation at least 3 days per week had the highest prevalence of self-reported recommended sleep duration (69% and 71%). Among black men and women, however, the highest prevalence of recommended sleep was among alcohol never-consumers (61% and 56%). Supplemental Figure S2 shows results by three, simplified alcohol consumption categories of never, moderate, and heavy.

#### 3.2.2. Black-White Differences in Short Sleep Duration by Sex

In fully-adjusted models, both black men and women were significantly more likely to report short sleep duration (<7 h) across all categories of alcohol consumption, compared to their white counterparts (Figure 2). There were significant interactions between sex and alcohol drinking patterns for short sleep (p_interaction_ = 0.01) among whites, but not among blacks (Supplemental Table S1, Supplemental Figure S3). We also did not find a significant interaction for drinking pattern by race for short sleep among men. Compared to white men who never consumed alcohol, short sleep was 25% (PR = 1.25 [95% CI: 1.14–1.37]) more prevalent among black men who never consumed alcohol. Among male drinkers and across consumption patterns, short sleep was also approximately 30% more prevalent among black men compared to their white counterparts. A significantly higher prevalence of short sleep was also observed among black women compared to white women across all categories of alcohol consumption. However, black women with moderate infrequent (1 drink/day ≤ 2 days/week), moderate frequent (1 drink/day 3–7 days/week), and heavy infrequent (≥2 drinks/day ≤ 2 days/week) consumption had a much higher prevalence of short sleep than their white counterparts, while the prevalence of short sleep was less disparate among black and white female never and heavy frequent-drinkers (≥2 drinks/day 3–7 days/week) (p_interaction_ = 0.004, Supplemental Table S1 and Supplemental Figure S4). Supplemental Figure S5 shows results by three, simplified alcohol consumption categories of never, moderate, and heavy.

#### 3.2.3. Black-White Differences in Long Sleep Duration by Sex 

Associations between alcohol consumption and long sleep varied by race in both sexes (p_interaction_ for men = 0.03 and p_interaction_ for women = 0.01, Supplemental Table S1). Among men, never-drinkers were more likely to be long sleepers among whites, and less likely to be long-sleepers among blacks (Supplemental Figure S4), but we observed the opposite among heavy drinkers. Compared to white men, black men reporting heavy infrequent drinking (consumed ≥3 drinks ≤ 2 days/week: PR = 1.30 [95% CI: 1.07–1.58]) or heavy frequent drinking (consumed ≥3 drinks 3–7 days/week: PR = 1.41 [95% CI: 1.12–1.76]) were more likely to report long sleep duration (Figure 2A). Among women, white heavy frequent drinkers had the highest prevalence of long sleep, but black moderate frequent drinkers had the highest prevalence of long sleep (Supplemental Figure S4). Only black women who reported moderate alcohol consumption (1 drink ≤ 2 days/week, PR = 1.16 [95% CI: 1.01–1.33] and 1 drink 3–7 days/week, PR = 1.67 [95% CI: 1.07–2.61]) had higher prevalence of long sleep than their white counterparts (Figure 2B).

#### 3.2.4. Black-White Differences in Sleep Quality by Sex

There were also racial differences in sleep quality for specific categories of alcohol consumption. Compared to white men, black men with moderate infrequent consumption (1–2 drinks ≤ 2 days/week) were significantly less likely to report trouble falling asleep (PR = 0.81 [95% CI: 0.71–0.92]) and trouble staying asleep (PR = 0.87 [95% CI: 0.76–0.98]), but black men with moderate frequent consumption (1–2 alcohol drinks 3–7 days/week) were more likely to have trouble staying asleep (PR = 1.24 [95% CI: 1.00–1.54]). Compared to white women, black women who never consumed alcohol and those with moderate infrequent alcohol consumption (1 drink ≤ 2 days/week) were significantly less likely to report trouble falling and staying asleep. Black men (PR = 0.61 [95% CI: 0.40–0.93]) and women (PR = 0.51 [95% CI: 0.43–0.62]) who never consumed alcohol were less likely than their white counterparts to report taking sleep medication at least once per week. Although a similar association was observed among male heavy frequent drinkers (PR = 0.60 [95% CI: 0.39–0.93]) black women had a lower prevalence of sleep medication usage than white women across all alcohol drinking patterns, except among heavy frequent drinkers (p_interaction_ = 0.0003, Supplemental Table S1 and Supplemental Figure S4) resulting in no racial difference in that group.

We conducted a sensitivity analysis excluding men and women under the age of 21 years and found no substantial change in the results for men. For women, we observed a significantly higher prevalence of long sleep among black women who never consumed alcohol (PR = 1.13 [95% CI: 1.02–1.25) or had ≥2 drinks ≤ 2 days/week (PR = 1.17 [95% CI: 1.04–1.32]). No substantial change in the PRs for other sleep duration and quality measures among women were observed.

The fitted restricted cubic models suggested a nonlinear association between alcohol drinking patterns and sleep duration among short, recommended, and long sleepers (Supplemental Figure S6). Among short sleepers, higher levels of alcohol consumption were associated with a steady decrease in sleep duration for white men and women with average alcohol consumption between 10 and 30 drinks per week. The overall curves for black men and, particularly, for women who were short sleepers were more variable with increasing alcohol consumption. Spline curves were much flatter for recommended and long sleepers, but greater variability sleep duration remained with increasing alcohol consumption among black men and women. 

## 4. Discussion

In a nationally representative sample of the US population, the relationship between alcohol drinking patterns and sleep duration differed between blacks and whites among both men and women. The groups most likely to report the recommended amount of sleep were blacks who never consumed alcohol and whites who frequently consumed in moderation. The prevalence of short sleep duration was higher among black men and women who consumed alcohol compared to their black counterparts who never consumed alcohol, but the prevalence of short sleep across alcohol consumption patterns was more variable among whites. Within each alcohol consumption category (whether never or excessive), blacks had a significantly higher prevalence of short sleep than whites. Interestingly, black-white differences in the prevalence of short sleep were greatest among participants who frequently drank alcohol in moderation. Alcohol consumption patterns and long sleep duration also varied by race-sex group. Racial differences in the relationship between alcohol consumption patterns and suboptimal sleep duration were more pronounced among women than men.

Alcohol use prior to falling asleep often leads to sleep disturbances [40]. In fact, alcohol consumption appears to have myriad effects on sleep architecture depending on chronic vs. acute usage, the dose, and timing of consumption. In short, alcohol can enhance sleep onset, but also decrease sleep continuity during the 2nd half of the sleep period. Additionally, alcohol has been shown to increase slow wave sleep (at least in the short-term) and suppress rapid-eye movement sleep [11,19,40]. Perpetual alcohol use disrupts sleep and large amounts for extended periods can degrade sleep quality [19,22]. In a prior study regarding alcoholism, alcoholic individuals had more sleep abnormalities than non-alcoholics, and black participants had more severe sleep abnormalities than whites [23]. Given these findings, it is surprising that we did not observe consistently higher prevalence of shorter sleep duration among white heavier drinkers.

Shorter sleep among blacks compared to whites could be due to greater exposure to stressful environments. Research suggests that chronic stress, such as institutional and interpersonal discrimination, is experienced more often by blacks and is associated with worse sleep and health outcomes among blacks [7,36,41,42]. While studies show an overall lower prevalence of alcohol use and abuse among blacks compared to whites [43], coping strategies in response to chronic stress may include unhealthy behaviors such as alcohol drinking [29]. Of note, chronic stress alone causes hypothalamic pituitary axis (HPA) dysregulation, is associated with altered circadian rhythms, and increases in circulating cortisol levels [44,45] while alcohol abuse is associated with greater sympathetic nervous system (SNS) activity among blacks, but with lower SNS activity among whites compared to non-alcohol abusers within racial groups [46]. Norepinephrine is associated with insomnia and increases in alertness, heart rate, and blood pressure [46], all of which are negatively associated with suboptimal sleep. Sleep deprivation, a result of habitual suboptimal sleep duration, is also hypothesized to elevate nocturnal cortisol over time [47]. If Black adults have HPA axis disruption due to chronic stress and sleep deprivation, the physiologic effects of alcohol use may further exacerbate racial disparities in sleep. Future longitudinal studies investigating these plausible biologic mechanisms and how they are affected by modifiable, environmental stressors are warranted.

The broader social environment may also contribute to racial differences in alcohol drinking patterns. For example, even though blacks compared to whites have lower rates of alcohol consumption and heavy drinking, black men especially, are more likely to encounter alcohol-related social consequences, such as legal problems because of drinking [27,28]. Potential social consequences due to alcohol drinking may negatively affect consumption behavior. In our study, white moderate and heavy drinkers had much higher income and education than their black counterparts, which agrees with prior literature stating—among blacks—heavier drinkers are often lower income and lack social capital [27]. The racial differences in drinkers illustrates that shorter and longer sleep durations observed among blacks (especially black males) compared to whites could be a result of stress and other social problems faced more often by lower socioeconomic status blacks compared to white moderate and heavy drinkers. This is an important area for further sleep research to disentangle race, sex, the social environment related to chronic stress, and alcohol use. 

Gene-environment interactions may also contribute. ADH1B*3, the most widely replicated gene variant of aldehyde dehydrogenase and the primary enzyme responsible for metabolizing alcohol, appears more prevalent among blacks than whites [26,46]. The ADH1B*3 allele is associated with significantly faster ethanol metabolism to acetaldehyde. Although ADH1B*3 appears protective against alcohol misuse disorders among blacks, it nonetheless may lead to adverse consequences among black carriers of the B*3 allele who drink [46]. Therefore, the racial disparities in suboptimal sleep duration we observed may also be a manifestation of the adverse health effects of alcohol consumption in the face of accelerated acetaldehyde production. Future research should investigate the potential role of gene-environment interactions.

Our results indicating race-sex interactions in the association between alcohol consumption and sleep are consistent with the prior literature. For example, in a study of 178 male and female young adults, there were race and gender differences in acute responses immediately following alcohol administration which remained after adjustment for recent alcohol use [48]. Pedersen and McCarthy examined the sedating and stimulating effects of alcohol on the ascending and descending limbs of blood alcohol curves after administering a dose of alcohol designed to make participants reach a peak blood alcohol concentration of 0.075 to 0.080 mg %. Men experienced sharper increased stimulation compared to women and blacks demonstrated greater stimulation compared to whites on the ascending, but not descending limbs of blood alcohol curves. Compared to white women, black women showed marginally increased sedation over time. Conversely, compared to white men, black men showed marginally slower sedation after alcohol administration. These observations highlight the complexity of the effects of alcohol consumption by race and sex. Nonetheless, it is plausible that in our large-scale epidemiological study, black men may have been more likely to have trouble staying asleep due to higher stimulation and less sedation than white men. Black women also may have longer sleep and fewer problems staying asleep due to increased alcohol-related sedation. 

Our study has limitations. For instance, we used a cross-sectional study design and temporality between alcohol consumption patterns and sleep duration/disturbances could not be established. Also, all data were based on self-report. Self-reported alcohol consumption data are reasonably valid and reliable even though the quantity may be underreported at levels above moderation [49,50,51,52]. Nonetheless, computerized assessments such as the computer-assisted personal interview used in the NHIS may overcome some of the disadvantages of self- and interviewer-administered instruments. Our measure of alcohol use was also based on the time period from the past year, but drinking patterns may change over time. Misclassification of sleep difficulties likely remains and objective measures of sleep quality may be necessary in future studies. For instance, blacks have been shown to be less likely to self-report sleep complaints (e.g. trouble falling and staying asleep) [53], but sleep is consistently worse than whites based on objective sleep measures [54]. Lastly, the observed association for alcohol and sleep could, in part, reflect unmeasured confounding or interactions of alcohol with unmeasured factors like psychosocial stress. Despite these limitations, this study has important strengths. First, the nationally representative nature of our data allows for inferences to the general US population of black and white adults. The sample size was large overall and in terms of racial/ethnic minority representation, which allowed for robust stratification of both race and sex. Second, we had greater detail on drinking patterns than is typically available. Third, we included absolute differences that are important for communicating impact and relative measures of association for the strength of the relationship while adjusting for potential confounders. Lastly, we included more recently collected data than prior studies.

## 5. Conclusions

Alcohol use is widespread in the US, which has important health implications. This study extends the public health and health disparities literatures by highlighting that its impact on sleep appears to differ by race and sex. Behavioral correlates like alcohol drinking patterns and sleep track together in a complex manner and could act in concert to contribute to health disparities by race and sex. Future studies on alcohol should include more racially-diverse participants. These studies should seek to understand the overall alcohol-sleep relationship and determinants of racial disparities, which may lead to novel identification of targets for interventions and mitigate downstream consequences as well as address health disparities. For instance, it would be useful to more closely investigate racial differences in the influence of drinking patterns on sleep. Given racial differences in consumption type and pattern, it would be equally interesting to study the reasons for drinking pattern differences as well as the impact of type of alcohol consumed on sleep. Future studies should also consider the influence of race-associated social and environmental stress on the relationship and potential future health impacts.

## Figures and Tables

**Table 1:** Sociodemographic, Health behavior, and clinical characteristics among US men by alcohol drinking patterns, National Health Interview Survey, 2004–2015 (*N* = 84,194).

Sociodemographic, Health Behavior, and Clinical Characteristics	Never Drinkers				Moderate Infrequent				Moderate Frequent				Heavy Infrequent				Heavy Frequent			
					1–2 Drinks ≤ 2 Days/Week				1–2 Drinks 3–7 Days/Week				≥3 Drinks ≤ 2 Days/Week				≥3 Drinks 3–7 Days/Week			
	White	%	Black	%	White	%	Black	%	White	%	Black	%	White	%	Black	%	White	%	Black	%
Sample size ^a^	9236	13.7	3184	26.7	27,485	38.8	5022	38.5	10,382	13.9	996	6.9	16,666	23.7	2553	19.9	7561	9.9	1109	8.0
Age, years, mean ± SE	45.2	0.31	38.3	0.44	48.8	0.16	42.4	0.32	54.0	0.23	46.2	0.61	37.4	0.18	38.0	0.36	44.5	0.28	43.2	0.53
Educational attainment																				
<High school	1192	13.1	576	19.3	1582	5.7	603	13.4	417	3.4	125	13.4	1108	9.0	350	18.0	573	8.7	213	20.3
High school graduate	3112	35.4	1307	41.5	6940	25.8	1718	34.9	1978	18.9	354	34.0	5362	34.8	974	37.5	2323	30.8	455	41.5
Some college	2557	26.6	841	24.7	8242	29.2	1678	32.4	2675	25.6	322	32.1	5957	32.9	861	32.3	2567	31.1	299	27.2
≥College	2306	24.9	435	14.5	10,669	39.2	1,001	19.4	5289	52.1	189	20.5	4213	23.3	362	12.2	2090	29.4	136	11.1
Marital status																				
Married	4705	59.2	980	45.0	16,182	69.6	1758	48.5	6447	72.0	318	43.0	6405	56.3	651	41.1	2828	52.8	279	38.9
Divorced/separated/widowed	1613	12.3	711	20.2	5688	14.7	1424	24.6	2218	13.9	350	30.4	3229	20.1	654	28.0	2131	24.7	363	30.3
Never married	2890	28.5	1487	34.8	5569	15.7	1825	26.9	1696	14.2	324	26.5	7007	23.6	1242	30.9	2594	22.5	463	30.8
Unemployed or not in workforce	4016	41.8	1548	51.2	8137	30.9	1837	42.2	3593	29.2	369	38.9	3391	33.0	833	43.6	2178	36.0	491	53.2
Annual Household income (<$35,000 per year)	3411	34.3	1616	50.1	6147	19.6	2142	39.2	1873	14.9	448	41.3	5385	28.1	1257	47.2	2631	28.6	638	52.9
Living in poverty	1096	9.9	637	18.6	1550	4.4	783	13.5	354	2.8	174	16.7	1804	7.2	494	19.3	790	8.1	267	23.9
Smoking status																				
Never	7047	75.2	2505	77.5	14,731	52.8	2827	52.8	4600	46.8	364	35.8	7139	35.8	1182	38.0	2302	28.2	343	28.9
Former	1267	14.6	275	10.5	8110	31.4	914	24.1	4172	37.3	221	26.1	3710	32.7	383	23.2	2111	33.3	185	22.0
Current	917	10.2	400	12.0	4625	15.8	1275	23.0	1603	15.8	410	38.1	5811	31.5	987	38.8	3144	38.5	580	49.0
Leisure-time physical activity ^b^																				
Never/unable	4078	45.2	1552	51.8	7111	26.4	1614	35.3	2131	19.5	361	36.9	3673	27.3	786	37.5	2019	29.0	488	44.3
Low	2350	25.9	773	23.9	10,109	37.3	1757	34.6	3711	37.0	279	29.2	6269	36.4	887	33.3	2512	33.5	287	25.9
High	2769	28.9	842	24.3	10,159	36.4	1635	30.2	4505	43.4	353	33.9	6672	36.3	871	29.2	3000	37.6	328	29.8
Sleep duration																				
<7 h	2143	23.1	912	28.2	7879	27.9	1993	38.5	2506	24.9	401	40.6	5403	29.4	1061	40.3	2391	29.6	450	38.7
7–<9 h	6090	66.3	1953	61.4	17,795	65.3	2633	52.5	7092	68.6	507	50.9	10,300	63.4	1266	49.2	4586	61.4	532	48.9
≥9 h	1003	10.6	319	10.5	1811	6.7	396	9.0	784	6.5	88	8.5	963	7.2	226	10.5	584	9.0	127	12.3
BMI, kg/m^2^, mean ± SE	27.5	0.07	27.9	0.11	28.0	0.04	28.3	0.09	26.9	0.05	27.6	0.18	28.2	0.06	28.4	0.14	27.3	0.07	27.1	0.20
Sad (past 30 days) (≥mostly)	215	2.3	85	2.2	466	1.6	171	2.9	133	1.1	39	3.8	352	2.1	80	2.9	248	3.0	70	5.8
Felt depressed (often/a lot)	71	13.7	15	12.9	215	10.5	54	15.0	64	10.4	13	15.8	186	15.1	22	9.5	77	9.7	10	14.7
Health outcomes																				
Hypertension	2861	33.0	1043	40.2	8807	34.1	1878	44.6	3665	33.2	412	43.4	3597	35.1	824	45.7	2475	39.6	451	45.6
Heart Disease	1311	14.6	249	9.5	3697	14.5	440	11.3	1597	13.4	93	10.5	1082	12.5	179	10.0	751	12.8	88	9.1
Cancer (yes)	963	10.3	133	4.9	2876	11.4	241	7.1	1488	12.6	59	8.5	646	9.1	64	5.1	603	12.3	40	5.0
Type 2 diabetes	1017	12.1	412	16.1	2439	9.8	633	16.4	582	5.1	93	10.6	662	8.6	211	13.4	320	5.7	87	10.0
Health status																				
Excellent/very good	5606	60.1	1731	49.2	18,180	65.7	2710	49.5	7452	74.0	524	50.9	11,608	62.1	1435	47.6	4767	59.9	498	41.7
Good	2319	25.7	861	28.8	6697	24.8	1468	32.0	2218	20.4	303	31.4	3870	27.8	765	35.0	2001	28.9	370	34.7
Fair/poor	1304	14.2	591	22.0	2600	9.5	844	18.5	709	5.6	169	17.7	1185	10.2	353	17.5	792	11.2	241	23.5
Region of residence																				
Northeast	1034	12.4	366	12.6	5104	20.3	600	13.2	2224	21.6	78	8.6	2773	20.1	278	12.9	1306	17.8	89	7.7
Midwest	2311	25.4	535	17.0	7737	28.3	971	19.2	2398	23.4	174	17.4	5857	34.7	510	19.0	2022	26.8	172	17.7
South	3837	43.3	2015	63.0	8574	32.1	2852	56.8	2956	30.2	583	59.8	4952	29.6	1532	59.4	2483	35.0	742	65.5
West	2054	19.0	268	7.4	6070	19.3	599	10.9	2804	24.9	161	14.2	3084	15.6	233	8.7	1750	20.4	106	9.1

Data presented as mean ± standard error or *n* (%) ^a^ Percentage may not sum to 100 due to missing values; SE = standard error; ^b^ Leisure-time physical activity = light or moderate leisure-time physical activity that lasts at least 10 min and that causes only light sweating or a slight to moderate increase in breathing or heart rate, and/or vigorous leisure-time physical activities that last at least 10 min and that causes heavy sweating or large increases in breathing or heart rate (Never = 0 times per week of light/moderate or vigorous leisure-time physical activities, Low = less than 1/week to 4 times/week of light/moderate or less than 1/week to 3 times/week of vigorous leisure-time physical activity, High = 5–28 times/week of light/moderate or 4–28 times/week of vigorous leisure-time physical activity). Heart disease = coronary heart disease or a heart condition.

**Table 2:** Sociodemographic, health behavior, and clinical characteristics among US women by alcohol drinking patterns, National Health Interview Survey, 2004–2015 (*N* = 103,756).

Sociodemographic, Health Behavior, and Clinical Characteristics	Never Drinkers				Moderate Infrequent				Moderate Frequent				Heavy Infrequent				Heavy Frequent			
					1 Drink ≤ 2 Days/Week				1 Drink 3–7 Days/Week				≥2 Drinks ≤ 2 Days/Week				≥2 Drinks 3–7 Days/Week			
	White	%	Black	%	White	%	Black	%	White	%	Black	%	White	%	Black	%	White	%	Black	%
Sample size ^a^	19,458	23.1	8671	42.6	24,656	29.4	4648	23.2	4904	5.6	303	1.4	28,058	34.7	6013	29.2	6256	7.2	789	3.4
Age, years, mean ± SE	52.5	0.26	44.4	0.31	50.8	0.15	43.7	0.31	56.5	0.32	50.3	1.21	39.8	0.14	38.5	0.26	48.4	0.29	42.7	0.58
Educational attainment																				
<High school	3102	13.4	1747	18.7	1056	4.1	383	8.5	100	1.6	35	11.3	1365	5.4	713	14.7	218	3.5	157	23.2
High school graduate	7231	37.1	3195	37.9	6121	25.6	1218	26.9	933	16.4	71	22.4	7083	30.2	1802	29.8	1254	21.5	233	31.4
Some college	5537	30.7	2545	29.7	8074	32.1	1782	37.0	1402	28.6	94	31.8	10,480	35.3	2379	36.7	2006	31.5	260	29.1
≥College	3499	18.8	1122	13.7	9359	38.2	1248	27.6	2464	53.4	102	34.6	9078	29.1	1098	18.8	2766	43.4	136	16.3
Marital status																				
Married	8437	55.8	1680	29.1	12,984	64.1	1084	33.7	2721	69.2	77	36.8	12,245	53.9	953	25.1	2818	57.4	117	26.0
Divorced/separated/widowed	7873	24.3	3391	35.0	8243	24.2	1638	36.9	1675	21.3	122	36.1	7508	28.4	1804	39.6	1973	25.2	282	41.6
Never married	3090	19.9	3553	35.9	3362	11.6	1904	29.5	500	9.5	103	27.1	8247	17.7	3236	35.3	1452	17.4	387	32.4
Unemployed or not in workforce	12,369	54.6	4711	54.0	10,371	41.0	1644	41.3	2297	38.6	120	40.9	7582	37.8	2046	44.4	2224	39.9	317	49.3
Annual Household income (<$35,000 per year)	9250	40.8	5613	61.5	6919	24.3	2229	44.6	969	15.5	139	41.3	9216	28.8	3502	54.2	1653	21.9	500	57.9
Living in poverty	2565	12.4	2706	29.9	1609	5.4	970	19.0	146	2.6	57	16.3	3434	7.9	1733	22.9	465	5.3	243	26.0
Smoking status																				
Never	15,407	79.6	7250	84.3	15,503	63.8	3208	65.9	2,546	54.4	138	47.5	13,665	45.7	3409	49.5	2376	37.1	278	33.2
Former	2126	9.6	599	6.9	5887	23.4	662	18.2	1854	35.2	61	21.4	6308	29.5	777	21.6	2065	36.3	119	22.3
Current	1906	10.8	815	8.7	3255	12.8	776	15.9	497	10.4	104	31.1	8068	24.8	1823	28.9	1811	26.6	392	44.4
Leisure-time physical activity ^b^																				
Never/unable	9965	48.2	5051	58.1	6530	26.4	1746	39.2	962	16.9	102	33.8	6217	25.9	2208	39.6	1277	20.8	358	45.8
Low	4464	24.9	1919	22.7	8850	37.1	1560	34.3	1666	36.1	99	31.9	11,007	38.2	2069	34.4	2150	34.9	192	24.4
High	4972	26.9	1680	19.2	9228	36.5	1334	26.5	2261	47.0	100	34.3	10,764	36.0	1727	26.0	2816	44.3	237	29.8
Sleep duration																				
<7 h	5035	25.0	2666	31.8	6730	26.7	1883	40.4	1087	22.2	126	36.2	8306	28.9	2504	41.5	1585	24.2	311	37.3
7–<9 h	12,007	63.6	4960	56.2	16,015	65.6	2408	50.8	3438	70.8	141	50.1	17,725	63.0	3011	49.9	4103	66.3	390	51.6
≥9 h	2416	11.5	1045	11.9	1911	7.6	357	8.8	379	7.0	36	13.7	2027	8.1	498	8.6	568	9.5	88	11.1
BMI, kg/m^2^, mean ± SE	27.1	0.06	29.3	0.08	26.9	0.05	29.62	0.12	24.4	0.08	27.33	0.36	26.75	0.05	29.63	0.13	25.11	0.07	28.17	0.26
Sad (past 30 days) (≥mostly)	696	3.6	354	3.9	638	2.4	195	3.3	67	1.2	19	4.6	820	2.6	356	5.2	177	2.5	83	9.4
Felt depressed (often/ a lot)	237	17.2	99	18.0	327	13.1	60	14.4	36	8.0	1	0.1	473	16.2	124	24.3	103	13.6	19	25.6
Health outcomes																				
Hypertension (yes)	7987	34.0	3879	47.9	7664	30.4	1848	47.9	1544	25.3	129	47.2	5363	29.6	2023	49.6	1682	28.7	320	55.4
Heart Disease (yes)	3097	12.4	882	10.8	2929	11.2	413	10.2	605	10.1	34	12.6	1978	9.4	394	9.3	521	9.4	74	11.3
Cancer (yes)	2671	10.7	441	5.2	3263	13.0	221	5.7	830	14.3	19	9.2	2123	11.4	214	6.2	754	13.8	39	5.8
Type 2 diabetes	2519	11.4	1311	17.6	1736	7.1	477	13.8	141	2.3	22	6.6	962	5.3	419	12.9	179	3.2	34	6.1
Health status																				
Excellent/very good	10,138	55.5	3882	42.6	16,334	66.7	2451	48.1	3769	78.5	166	56.8	19,729	67.3	3211	46.5	4592	72.5	377	42.1
Good	5595	26.8	2744	32.7	6093	24.8	1456	34.2	862	16.9	98	31.0	6342	24.5	1839	33.4	1255	20.8	258	36.7
Fair/poor	3713	17.7	2042	24.7	2219	8.5	738	17.8	272	4.7	39	12.1	1979	8.2	962	20.1	406	6.7	154	21.1
Region of residence																				
Northeast	2274	12.4	975	11.6	4830	20.7	608	14.7	1131	22.6	41	13.2	5206	21.3	882	16.7	1250	20.2	110	15.7
Midwest	4936	25.2	1480	16.3	7068	28.8	858	19.5	1039	21.2	48	17.0	9030	31.6	1254	22.5	1319	21.9	132	16.3
South	8820	46.8	5649	66.2	7225	30.6	2711	56.5	1412	30.1	166	55.3	8500	30.4	3338	52.4	1947	31.9	470	57.5
West	3428	15.6	567	5.9	5533	19.9	471	9.3	1322	26.1	48	14.4	5322	16.7	539	8.4	1740	26.0	77	10.5

Data presented as mean ± standard error or *n* (%); ^a^ Percentage may not sum to 100 due to missing values; SE = standard error; ^b^ Leisure-time physical activity = light or moderate leisure-time physical activity that lasts at least 10 min and that causes only light sweating or a slight to moderate increase in breathing or heart rate, and/or vigorous leisure-time physical activities that last at least 10 min and that causes heavy sweating or large increases in breathing or heart rate (Never = 0 times per week of light/moderate or vigorous leisure-time physical activities, Low = less than 1/week to 4 times/week of light/moderate or less than 1/week to 3 times/week of vigorous leisure-time physical activity, High = 5–28 times/week of light/moderate or 4–28 times/week of vigorous leisure-time physical activity). Heart disease = coronary heart disease or a heart condition.

## Supplementary Materials

The following are available online at http://www.mdpi.com/1660-4601/15/3/557/s1. Table S1: Interactions between (A) sex and alcohol drinking pattern stratified by race (B) race and alcohol drinking pattern stratified by sex, for sleep duration and sleep quality, National Health Interview Survey, 2004–2015, Figure S1: composition of analytic sample, Figure S2: Age-standardized proportions across alcohol drinking pattern for men (A) and women (B) by race over sleep duration categories, Figure S3: Interaction between sex and alcohol drinking pattern for sleep duration and sleep quality among White and Black, National Health Interview Survey, 2004–2015, Figure S4: Interaction between race and alcohol drinking pattern for sleep duration and sleep quality among male and female, National Health Interview Survey, 2004–2015, Figure S5: Fully-adjusted prevalence ratios for sleep duration and quality in relation to alcohol consumption among U.S. black men (a) and women (b) (referent white men or white women), national health interview survey, 2004–2015. Figure S6: Relationships between (A) short, (B) recommended and (C) long sleep durations and average number of alcohol drinks per week among U.S. black men and women stratified by race, National Health Interview Survey, 2004–2015.
