# Supplementary material for: The Relationship between Alcohol Drinking Patterns and Sleep Duration among Black and White Men and Women in the United States
Source: Int J Environ Res Public Health. 2018 Mar 20;15(3):557. doi: 10.3390/ijerph15030557 (PMC5877102; doi:10.3390/ijerph15030557)
Supplement: Supplementary file 1 [file ijerph-15-00557-s001.docx]

**Supplemental Materials**

NHIS, 2004-2015

(N=1,127,615)

Not Sampled (by design)/no record (N=768,048)

0

Not NH White or NH Black (N=89,135)

Not US born (N=15,419)

Invalid body weight (N=23,938)

Not NH White or

NH Black (N=231,075)

Eligible Sample Adults

(N=231,075)

Missing or invalid sleep data (N=2,849)

Missing or invalid alcohol data (N=40,276)

Final Analytic (N=187,950)

Not NH White or NH Black (N=89,135)

27)

**Figure S1.** Composition of Analytic Sample.

**Table S1.** Interactions between (A) sex and alcohol drinking pattern stratified by race (B) race and alcohol drinking pattern stratified by sex, for sleep duration and sleep quality, National Health Interview Survey, 2004-2015.

|  | 1. **Alcohol drinking pattern I^a^** | | 1. **Alcohol drinking pattern II^b^** | |
| --- | --- | --- | --- | --- |
|  | ***P* for interaction** | | ***P* for interaction** | |
| **Sleep duration** | **White** | **Black** | **Male** | **Female** |
| <7 hours vs. 7-8 hours | **0.01** | 0.10 | 0.09 | **0.0004** |
| ≥ 9 hours vs. 7-8 hours | 0.21 | 0.22 | **0.03** | **0.01** |
| **Trouble falling asleep (yes)*** | 0.13 | 0.61 | 0.34 | 0.05 |
| **Trouble staying asleep (yes)*** | 0.47 | 0.31 | 0.06 | 0.09 |
| **Days woke up feeling rested (most)*** | 0.09 | 0.08 | 0.15 | 0.13 |
| **Times took sleep medication last week ≥ 1** | **0.005** | 0.13 | 0.42 | **0.0003** |

^a^Alcohol drinking pattern I is defined as never, moderate, and heavy. For men, moderate drinking is defined as 1-2 drinks ≤ 2 days/week, heavy drinking is defined as ≥1 drink 3-7 days/week or ≥3 drinks≤ 2 days/week. For women, moderate drinking is defined as 1 drink ≤ 2 days/week, heavy drinking is defined as ≥1 drink 3-7 days/week or ≥2 drinks≤ 2 days/week.

^b^Alcohol drinking pattern II is defined as, for men: never, 1-2 drinks ≤2 days/week, 1-2 drinks 3-7 days/week, ≥3 drinks ≤2 days/week, and ≥3 drinks 3-7 days/week; for women: never, 1 drink ≤2 days/week, 1 drink 3-7 days/week, ≥2 drinks ≤2 days/week, and ≥2 drinks 3-7 days/week.


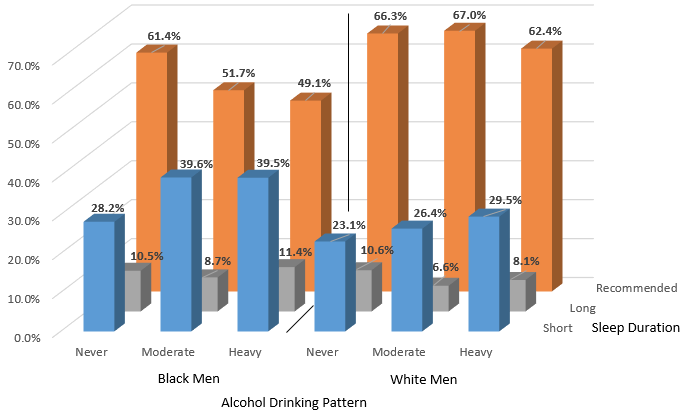


(**A**)


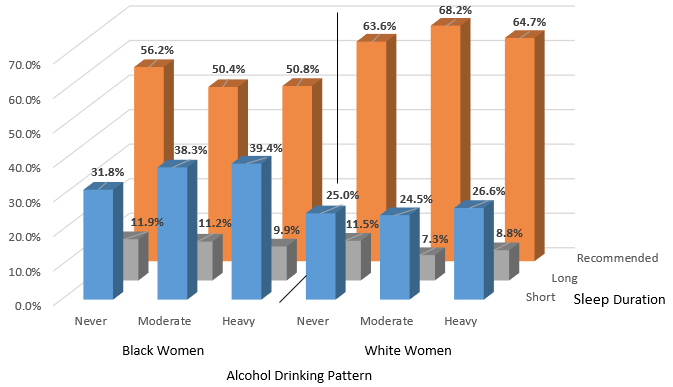


(**B**)

**Figure S2.** Age-standardized proportions across alcohol drinking pattern for men (A) and women (B) by race over sleep duration categories. Note: Alcohol drinking pattern for men: never= never drinkers; moderate=1-2 drinks ≤2 days/week and 1-2 drinks 3-7 days/week; heavy= ≥3 drinks ≤2 days/week and ≥3 drinks 3-7 days/week. Sleep duration: short=<7 hours; long=≥9 hours, recommended= 7-<9 hours. Alcohol drinking pattern for women: never= never drinkers; moderate= 1 drink ≤2 days/week and 1 drink 3-7 days/week; heavy= ≥2 drinks ≤2 days/week and ≥2 drinks 3-7 days/week. Sleep duration: short: <7 hours; long: ≥9 hours, recommended: 7-<9 hours.

| 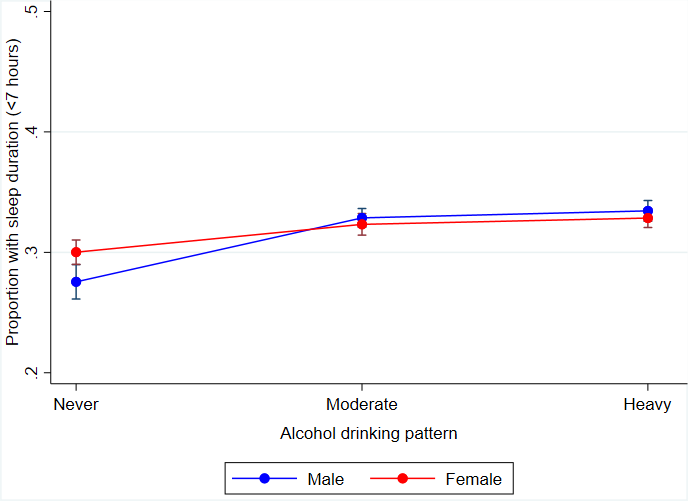  White: Short Sleep* | 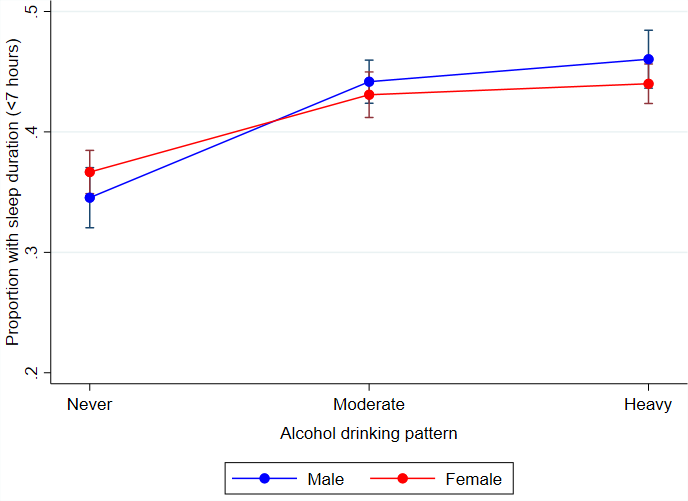  Black: Short Sleep |
| --- | --- |

(**A**)

| 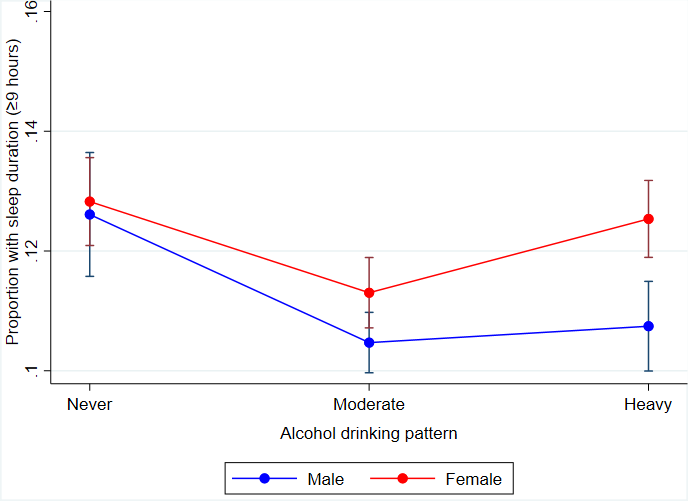  White: Long Sleep | 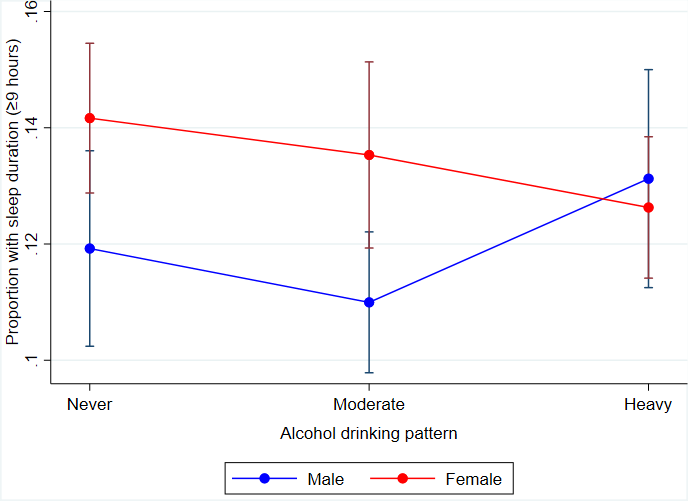  Black: Long Sleep* |
| --- | --- |

(**B**)

| 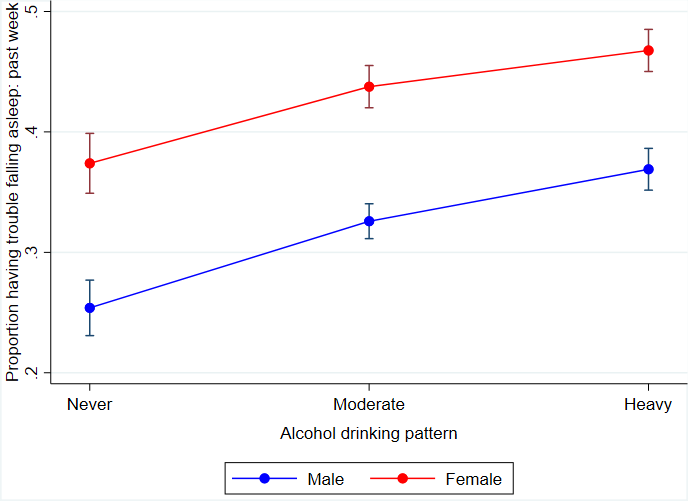  White: Trouble falling asleep* ^a^ | 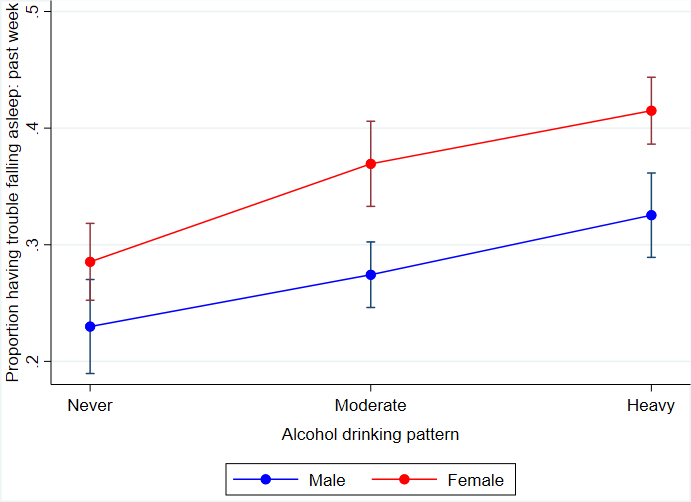  Black: Trouble falling asleep ^a^ |
| --- | --- |

(**C**)

| 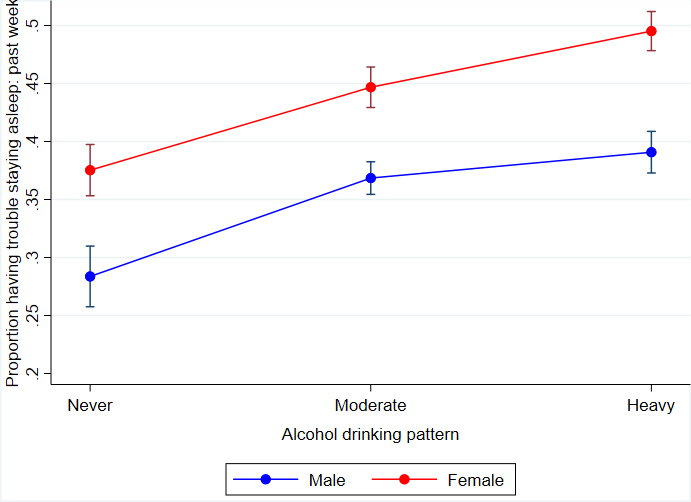  White: Trouble staying asleep ^b^ | 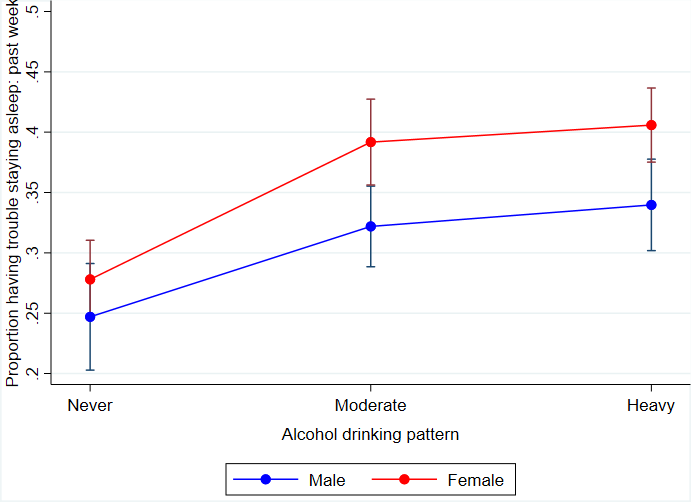  Black: Trouble staying asleep ^b^ |
| --- | --- |

(**D**)

| 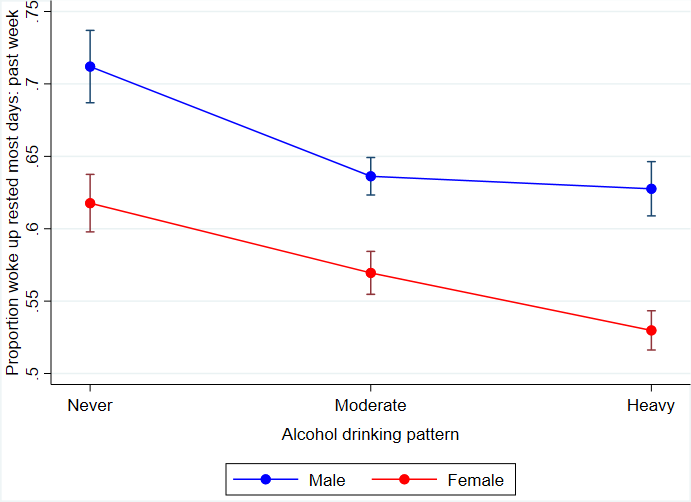  White: Days woke up feeling rested* ^c^ | 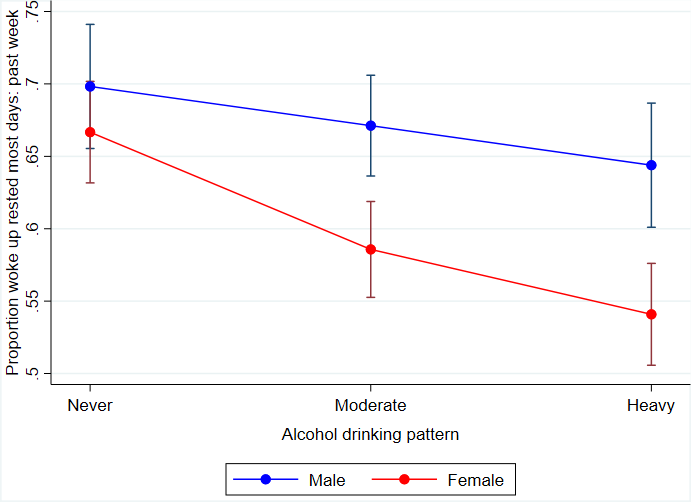  Black: Days woke up feeling rested ^c^ |
| --- | --- |

(**E**)

| 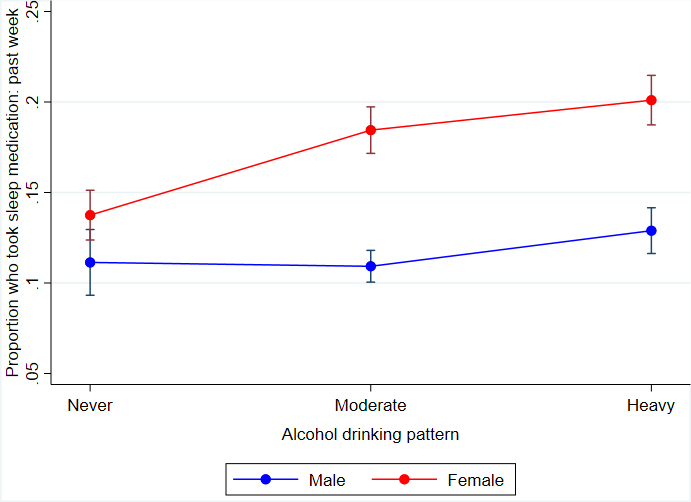  White: Times took sleep medication past week* ^d^ | 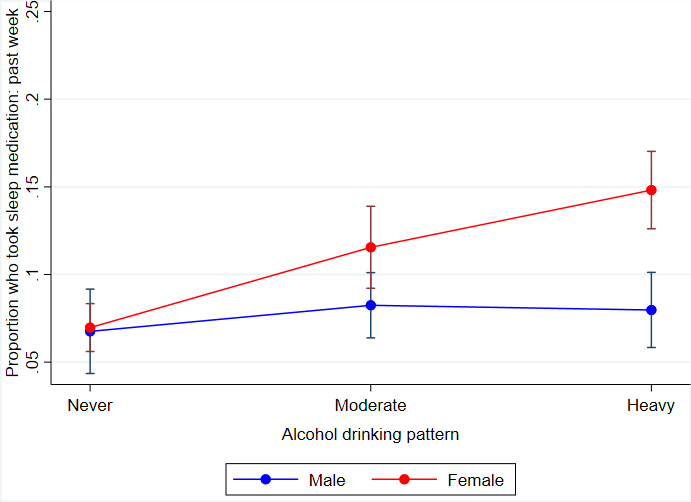  Black: Times took sleep medication past week* ^d^ |
| --- | --- |

(**F**)

**Figure S3.** Interaction between sex and alcohol drinking pattern for sleep duration and sleep quality among White and Black males and females, National Health Interview Survey, 2004-2015. Note: ^a^ Number of times having trouble falling asleep over the past week (1-7 or more times vs. never); ^b^ Number of times having trouble staying asleep over the past week (1-7 or more times vs. never); ^c^ Days woke up feeling rested over the past week (4-7 days vs. 0-3 days); ^d^ Number of times taking medication for sleep over the past week (1-7 or more days vs. never).

|   Male: Short Sleep |   Female: Short Sleep * |
| --- | --- |

(**A**)

| ****  Male: Long Sleep * | ****  Female: Long Sleep * |
| --- | --- |

(**B**)

| ****  Male: Trouble falling asleep ^a^ | ****  Female: Trouble falling asleep ^a^* |
| --- | --- |

(**C**)

| ****  Male: Trouble staying asleep ^b^ | ****  Female: Trouble staying asleep ^b^ |
| --- | --- |

(**D**)

| ****  Male: Days woke up feeling rested ^c^ | ****  Female: Days woke up feeling rested ^c^ |
| --- | --- |

(**E**)

| ****  Male: Times took sleep medication in past week ^d^ | ****  Female: Times took sleep medication in past week ^d^ |
| --- | --- |

(**F**)

**Figure S4.** Interaction between race and alcohol drinking pattern for sleep duration and sleep quality among males and females, National Health Interview Survey, 2004-2015. Note: ^a^Number of times having trouble falling asleep over the past week (1-7 or more times vs. never); ^b^Number of times having trouble staying asleep over the past week (1-7 or more times vs. never); ^c^Days woke up feeling rested over the past week (4-7 days vs. 0-3 days); ^d^Number of times taking medication for sleep over the past week (1-7 or more days vs. never); *Significant interactions.


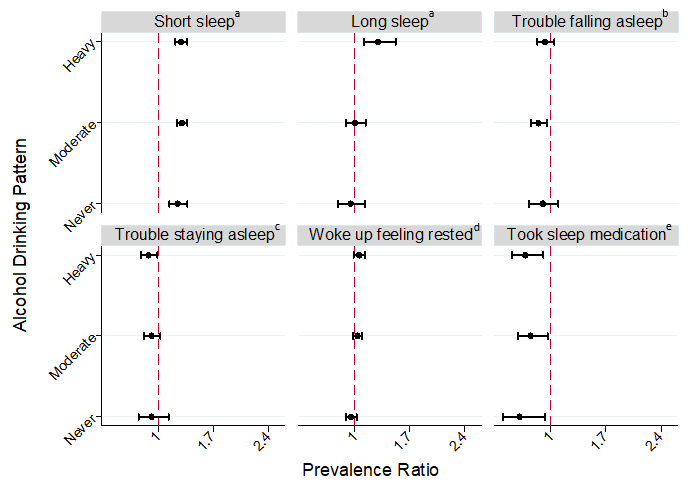


(**A**)


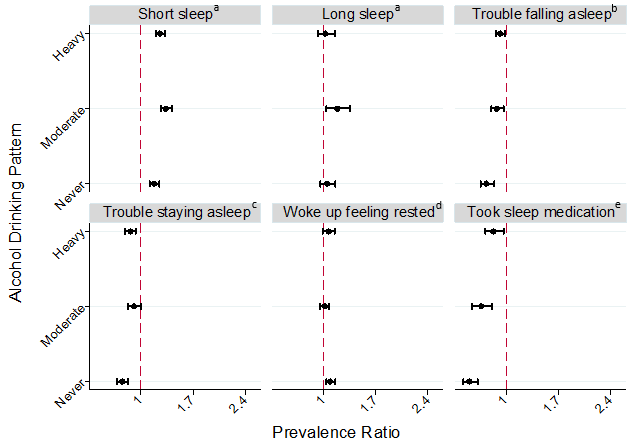


(**B**)

**Figure S5.** Fully-Adjusted Prevalence Ratios for Sleep Duration and Quality in Relation to Alcohol Drinking Patterns among U.S. Black Men (A) and Women (B) (referent: White Men or White Women), National Health Interview Survey, 2004-2015. Note. Short sleep: <7 hours; long sleep: ≥9 hours. Prevalence ratios adjusted for age, BMI, educational attainment, income, employment status, smoking, physical activity, diabetes, hypertension, heart disease, cancer, feeling sad (past 30 days), health status, and region of residence. Sleep quality data is from 2013-1015. ^a^ The referent category is white men (A) or white women (B) with recommended sleep (7-<9 hours). ^b^ Number of times having trouble falling asleep over the past week (1-7 or more times vs. never) ^c^ Number of times having trouble staying asleep over the past week (1-7 or more times vs. never). ^d^ Days woke up feeling rested over the past week (4-7 days vs. 0-3 days). ^e^ Number of times taking medication for sleep over the past week (1-7 or more days vs. never).* (A) Significant interactions between race and alcohol drinking pattern for men (long sleep: p_interaction_=0.01); (B) Significant interactions between race and alcohol drinking pattern for women (short sleep: p_interaction_=0.00001; trouble falling asleep: p_interaction_=0.03; took sleep medication: p_interaction_=0.0005).


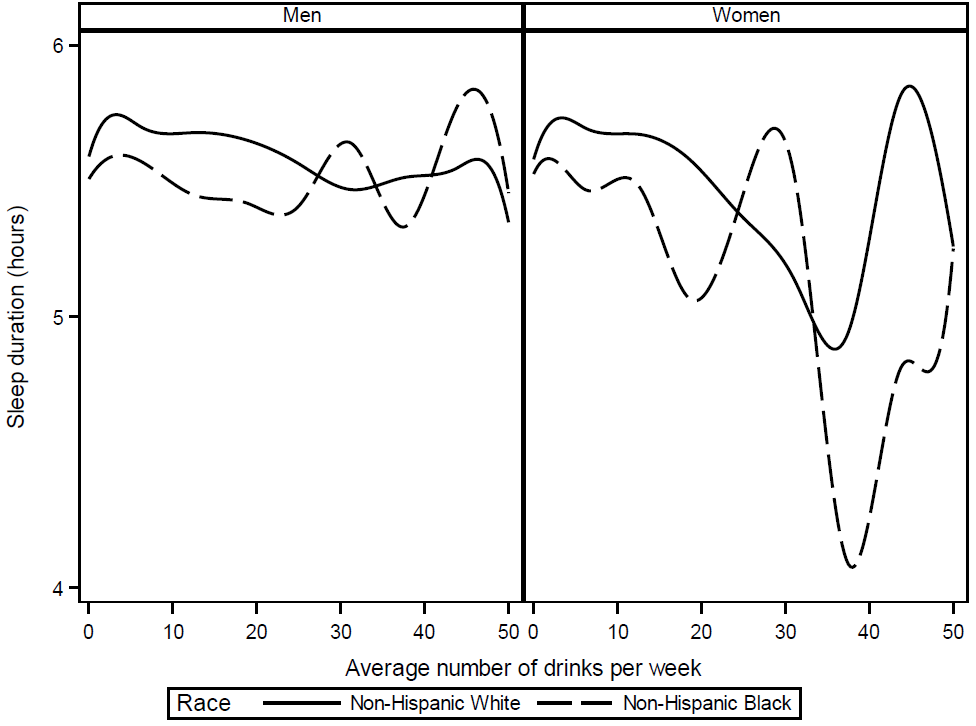


(**A**)

**
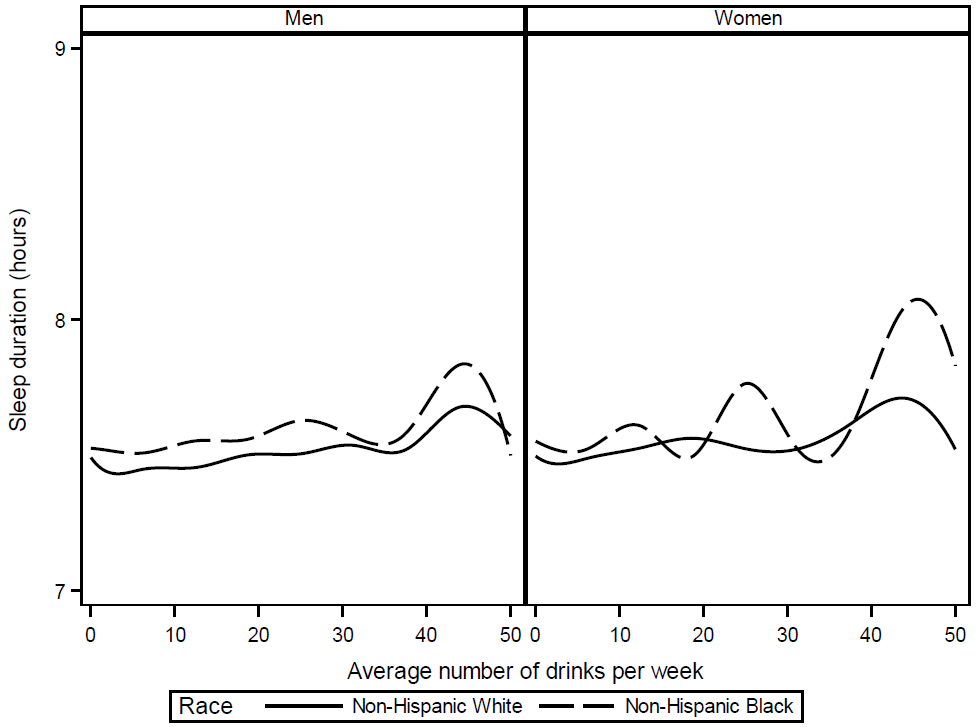
.**

(**B**)


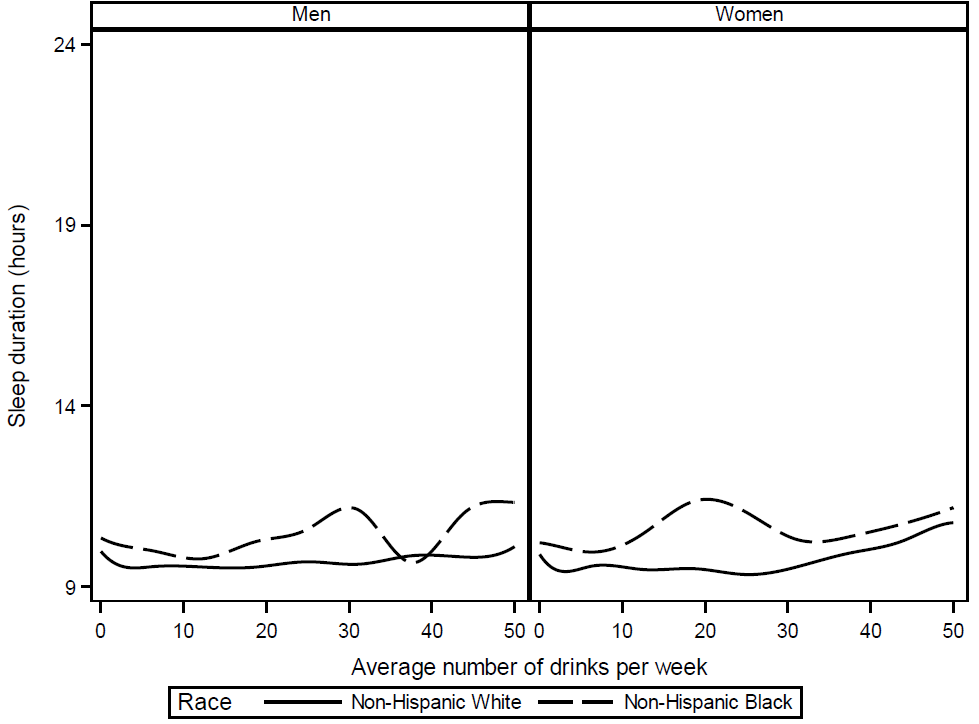


(**C**)

**Figure S6.** Relationships between (A) short, (B) recommended and (C) long sleep durations and average number of alcohol drinks per week among U.S. black men and women stratified by race, National Health Interview Survey, 2004-2015. Note: A) Short sleep: <7 hours; B) recommended sleep (7-<9 hours), C) long sleep: ≥9 hours. All models adjusted for age, BMI, educational attainment, income, employment status, smoking, physical activity, and region of residence.
